# Expression of GABA_Aα1_, GABA_B1_, and mGluR2 receptors in the lateral geniculate body of male neonates born to diabetic rats

**DOI:** 10.22038/IJBMS.2023.69668.15171

**Published:** 2023

**Authors:** Nasim Alipour, Somaye Fallahnezhad, Javad Bagheri, Hamideh Babaloo, Fatemeh Tahmasebi, Ghasem Sazegar, Hossein Haghir

**Affiliations:** 1 Department of Anatomy and Cell Biology, School of Medicine, Mashhad University of Medical Sciences, Mashhad, Iran; 2 Nervous System Stem Cell Research Center, Semnan University of Medical Sciences, Semnan, Iran; 3 Department of Anatomical Sciences, School of Medicine, Semnan University of Medical Sciences, Semnan, Iran; 4 Regenerative Medicine, Organ Procurement and transplantation Multidisciplinary Center, Razi Hospital, School of Medicine, Guilan University of Medical Sciences, Rasht, Iran; 5 Medical Genetic Research Center (MGRC), School of Medicine, Mashhad University of Medical Sciences, Mashhad, Iran

**Keywords:** Diabetes mellitus, Gamma-aminobutyric acid, Geniculate bodies, Glutamate metabotropic – receptors, Metabotropic glutamate

## Abstract

**Objective(s):**

Diabetes during gestation is one of the most common pregnancy complications and has adverse effects on offspring, including a negative impact on the offspring’s central nervous system (CNS). Diabetes is a metabolic disease associated with visual impairment. Due to the importance of the lateral geniculate body (LGB) in the visual pathway, the present study examined the effect of maternal diabetes on the expression of gamma-aminobutyric acid (GABA_Aα1_ and GABA_B1_) and metabotropic Glutamate (mGlu2) receptors in the LGB of male neonates of diabetic rats.

**Materials and Methods:**

Diabetes was induced in female adult rats by a single intraperitoneal dose of streptozotocin (STZ) 65 (mg/kg). In the Insulin-treated diabetic rats, diabetes was controlled by subcutaneous NPH-insulin injection daily. After mating and delivery, male offspring were killed by carbon dioxide gas inhalation at P0, P7, and P14 (postnatal days 0, 7, and 14). The expression of GABA_Aα1_, GABA_B1_, and mGluR2 in the LGB of male neonates was determined using the immunohistochemistry (IHC) method.

**Results:**

The expression of GABA_Aα1_ and GABA_B1_ was significantly reduced, whereas the expression of mGluR2 was markedly increased in the diabetic group compared with the control and insulin-treated groups at P0, P7, and P14.

**Conclusion:**

The results of the present study showed that induction of diabetes altered the expression of GABA_Aα1_, GABA_B1_, and mGluR2 in the LGB of male neonates born to diabetic rats at P0, P7, and P14. Moreover, insulin treatment could reverse these effects of diabetes.

## Introduction

Diabetes during pregnancy is associated with an increased risk of neurological disorders in offspring (1). Among the complications of diabetes, visual disturbances, and vision loss are of particular importance (2). The subcortical visual system consists of a large group of nuclei including the lateral geniculate body (LGB) (3). LGB is a structure located in the metathalamus and is responsible for the connection between the optic nerve and the primary visual cortex. LGB nuclei are divided into a dorsal and a ventral part. Both of these subsets receive signals from retinal ganglion cells that are transmitted by the optic nerve, however, only the dorsal part transmits the signals to the visual neocortex (4, 5). LGB contains different neurotransmitters (6) and its function depends on tight control of these excitatory/inhibitory neurotransmitter levels. The impaired balance of excitatory/inhibitory neurotransmitters in the neural network plays an important role in the pathophysiology of a wide variety of neurodevelopmental disorders (7). Gamma-aminobutyric acid (GABA) is the main inhibitory neurotransmitter in the central nervous system (CNS) (8, 9). GABA receptors are present throughout the visual pathway (10). Two structural types of GABA receptors including GABA_A_ and GABA_C_ are ionotropic but the GABA_B _receptors are metabotropic. GABA_A_ consists of 19 subunits that are encoded by different genes, including α, β, δ, ϒ, ε, and σ. Subunits α_1_ and ϒ_2_ have the highest expression among all subunits. The GABA_B_ receptors include GABA_B1a_, GABA_B1b_, and GABA_B2_. (11, 12). GABA receptors are present in LGB at the early postnatal stages. The high-affinity uptake of GABA displayed a noticeable peak on a postnatal day 15 in rat LGB (6). GABA receptor subunits including GABA_Aα1_ and GABA_B_, and Glutamic acid decarboxylase (GAD) expression significantly decreased in diabetic rats’ cerebral cortex (13). Glutamate is the main excitatory neurotransmitter in the mammalian CNS including LGB (6, 14). Glutamate receptors are divided into two groups, including ionotropic and metabotropic receptors (15). There are eight types of metabotropic receptors (mGluR_1_-mGluR_8_) that belong to the G protein-coupled receptor superfamily (GPCRs), which are classified into three groups (16). Glutamate expression increases in the visual cortex and LGB after birth and reaches the activity level of this receptor in adults 15 to 20 days after birth (6). 

Group II mGluRs are found in the thalamus (and LGB as a thalamic nucleus) and are main to circuit behavior in this structure, group II mGluRs modulate the thalamic relay to the cortex. Postsynaptic mGluR subtypes exhibit different distributions and may have clear functional roles in the visual system. Thalamocortical neurons in the LGB dynamically transmit visual signals from the retina to the neocortex, and this function can be modulated by the activation of mGluRs. glutamate receptors (mGluRs), which can be activated by retinogeniculate and corticothalamic pathways in the LGB (17).

Liu Z. *et al*. (2019) reported that diabetes is associated with abnormalities in glutamate receptors in the brain that cause neuronal degeneration (18). Diabetes can affect the central visual system, including LGB, in rats (19). The effects of diabetes on the central visual system are due to high blood glucose. High blood glucose of a diabetic mother easily enters the fetus through the placenta during pregnancy, so it is reasonable to imagine that it can affect the developing nervous system of the fetus too. Since the effects of maternal diabetes on the expression of neurotransmitter receptors involving the visual system of neonates have not been studied yet, we decided to investigate the distribution pattern of GABA_Aα1_, GABA_B1,_ and mGluR2 receptors using the IHC technique in the LGB of male rat neonates born to diabetic mothers.

## Materials and Methods

All experiments involving animals were accomplished by the guidelines of the National Institutes of Health (NIH) and accepted by the Committee for the Use of Laboratory Animals in Mashhad University of Medical Sciences (MUMS), Mashhad, Iran (IR.MUMS.medical.REC.1398.794).

***Animals***

Female Wistar rats (body weight: 200-250 g; age range: 6-8 weeks) were obtained from the animal house of Mashhad University of Medical Sciences (Mashhad, Iran). Animals were maintained in a room at 23±2 ^°^C (55±5% humidity, with a 12 hr light/12 hr darkness cycle) with standard water and feed. Two female rats in the same group were kept in a cage. The animals (n=15) were randomly divided into three groups: 1. Control (Con; n=5), 2. Diabetic (Dia; n=5), 3. Insulin-treated diabetic (Ins; n=5).

***Diabetes induction***

Diabetes was induced by an intraperitoneal injection of a single dose (65 mg /kg) of streptozotocin (STZ, Sigma Aldrich, Hamburg, Germany) diluted in normal saline in both the Dia and the Ins groups (20). The fasting blood glucose was measured by tail blood sampling 72 hr after STZ injection by a commercial digital glucometer (Accu-chek®, Germany). The rats with blood glucose levels of more than 150 mg/dl were considered diabetic and were divided into two groups, including Dia and Ins (21). In the Dia group, the blood glucose level was measured by collecting blood from the end of the caudal vein daily, the mean blood glucose level in the Dia group was 358.3±84.24 mg/dl, in addition, signs of binge drinking were observed in the rats of this group. The rats in the Ins group received 2-4 units of protamine-zinc insulin (NPH) (Exir Pharmaceutical Company, Iran) subcutaneously twice a day after becoming diabetic (22). Injectable insulin doses were determined by measuring daily blood glucose to make sure that the rats’ blood glucose levels were kept within the normal range. The mean blood glucose level in the Ins group was 105.3±10.14 mg/dl. To eliminate the potential effect of injection, the Con group rats received intraperitoneal injections of normal saline. Female rats in all three groups were caged with healthy male rats (body weight: 250–300 g; age range: 16 weeks), one week after developing diabetes in the Dia group and after diabetes control in the Ins group. Animals were allowed to give birth naturally at the end of the pregnancy period; the birthday was considered P0 (23). Rat pups born to diabetic and insulin-treated diabetic mothers were fed by healthy mothers to eliminate the possible effects of milk from diabetic rats and thus focus only on the fetal period environment (24). Male neonates in each group were randomly divided into three subgroups: P0 (n=5), P7 (n=5), and P14 (n=5).

***Tissue preparation***

Male neonates at the P0, P7, and P14 subgroups were sacrificed using CO_2_ inhalation on postnatal days 0, 7, and 14, respectively. The animal brains were removed from the skull and fixed in a 10% formalin solution for 72 hr. After fixation, the brains were dehydrated using ascending concentration ethanol (70 to 100%) and then immersed in paraffin. Coronal brain sections including LGB were prepared with a thickness of five micrometers.

***Immunohistochemistry***

ScyTek-ACV999 kit was used to perform immunohistochemistry (for details see below). The LGB sections were deparaffinized using xylene. The sections were hydrated using descending ethanols (100 to 70%), then washed with 0.1 M phosphate-buffered saline (PBS). Antigen retrieval was performed using Tris Buffered Saline 1% (TBS 1X, T5912-Sigma) at 96 ^°^C for 15 min, and then washed with PBS three times.

To inhibit endogenous peroxidase, the sections were treated with 3% hydrogen peroxide (Sigma-7722-84-1) in methanol for 10 min, and then the slides were washed three times with PBS. Subsequently, the sections were incubated overnight at 4 ^°^C with the primary antibody diluted in PBS. Primary antibodies included: 

GABABR1 antibody: orb85203, GABA A Receptor alpha 1 antibody: orb10677, MGLUR2 antibody: orb85332.

Then, slides were washed with PBS and incubated with 100 µl from Linker (Diagnostic BioSystems-PVP1000D) for 15 min. In the next stage, the sections of LGB were incubated with DAB (3, 3′-Diaminobenzidine) solution (ScyTek-ACV999), then washed with water. The sections were counterstained with Harris hematoxylin for 10 sec, then were again washed with water, dehydrated using ascending ethanol, clarified with Xylene, and finally, covered with coverslips (25, 26).

***Quantitative analysis***

**Figure 1.**
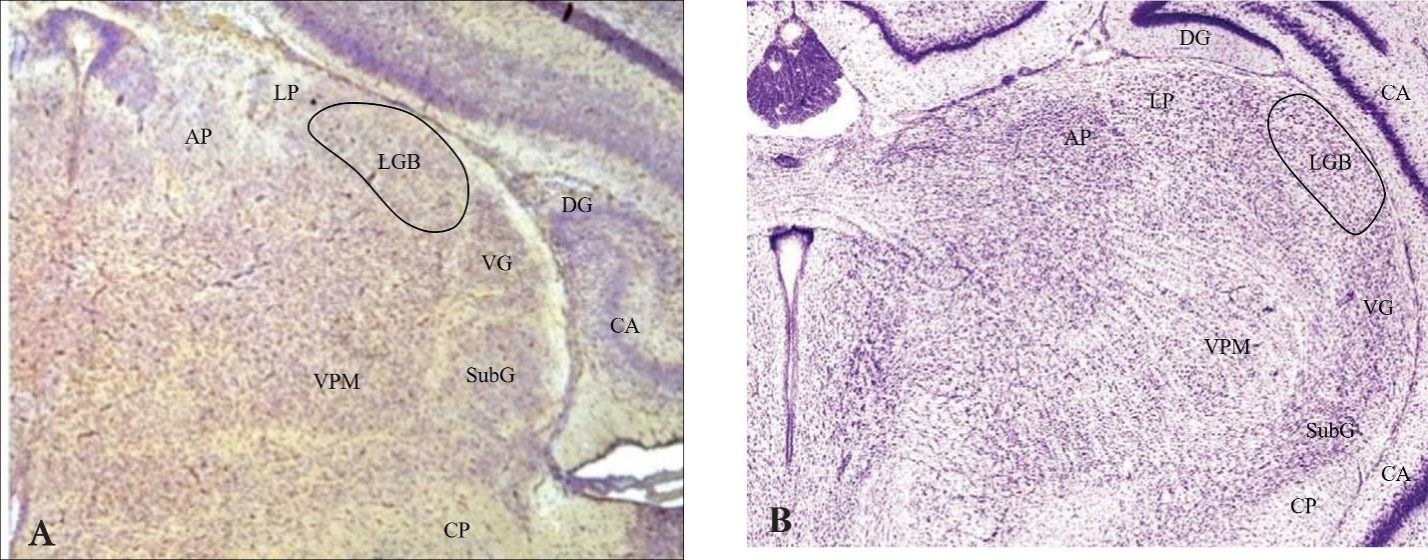
(A) Coronal section of a brain neonate (line curve shows the LGB boundary) that corresponds to the cross-section of the rat brain atlas (B)

Tissue sections were examined by means of an optical microscope (Olympus BX51, Japan) linked to a camera (Olympus DP12, Japan) by ND25 and OP filters. LGB boundaries in rat neonates were delineated by cross-referencing with the classical atlases of the rat brain (26-28) (Figure 1). Quantitative analysis of the immunohistochemical staining intensity in LGB was evaluated by ImageJ software. Using the subsequent formula calculated optical density (OD) for further statistical analysis.

Optical Density = log (Max intensity/Mean intensity)

The maximum intensity in Red-Green-Blue images is 255 and the mean intensity was measured by ImageJ software (18, 29).

***Statistical analysis***

Data were analyzed using GraphPad Prism 9 software, and they were compared through one- and two-way ANOVA, as well as *post hoc* Tukey’s test. All data were expressed as mean±SEM. A *P*<0.05 was considered statistically significant.

## Results

***GABA***_B1_ ***expression in LGB***

**Figure 2:**
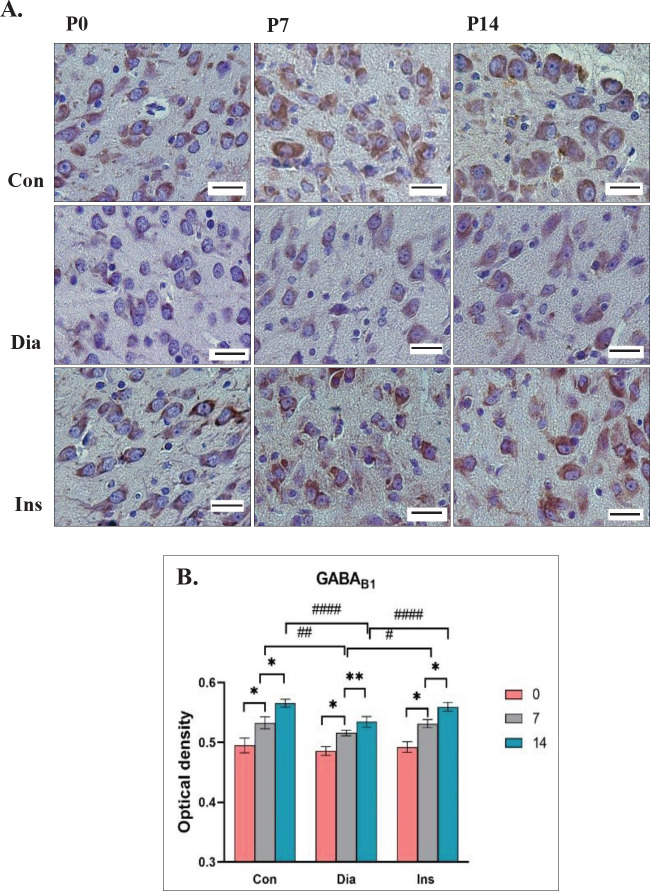
Immunoreactivity of the GABAB1 in the Lateral Geniculate Body (LGB) which has been displayed by the brown color is different on different days at P0, P7, and P14 in different groups (Con: neonates of control rats, Dia: neonates of diabetic rats, Ins: neonates of diabetic rats treated with insulin)

In the LGB, the main effect on the expression of GABA_B1 _in treatment groups was significantly different [F (2, 36)=22.21, *P*<0.0001]. The expression of GABA_B1_ was significantly decreased at P7 and P14 in the Dia group compared with the Con group (*P*<0.01 and *P*<0.0001, respectively). In the Ins group, the expression of GABA_B1_ significantly increased compared with the Dia group at P7 and P14 (*P*<0.05 and *P*<0.0001, respectively). No remarkable difference was perceived in the expression of this receptor in the LGB between rat neonates of the Ins and the Con groups at P0, P7, and P14. The main effect was significantly different in postnatal days [F (2, 36)=209.0, *P*<0.0001]. In all three groups, the OD of GABA_B1_ receptor expression in the LGB significantly increased from P0 to P7 and from P7 to P14 (each, *P*<0.0001). A significant difference was not seen in the main interaction effect of treatment groups×postnatal days [F (4, 36)=2.593, *P*=0.0527]. The highest OD was for the Con group at P14 (0.5310±0.02045), and the lowest OD was for the Dia group at P0 (0.5119±0.01421) (Figure 2).

***GABA***_Aα1_ ***expression in the LGB***

**Figure 3:**
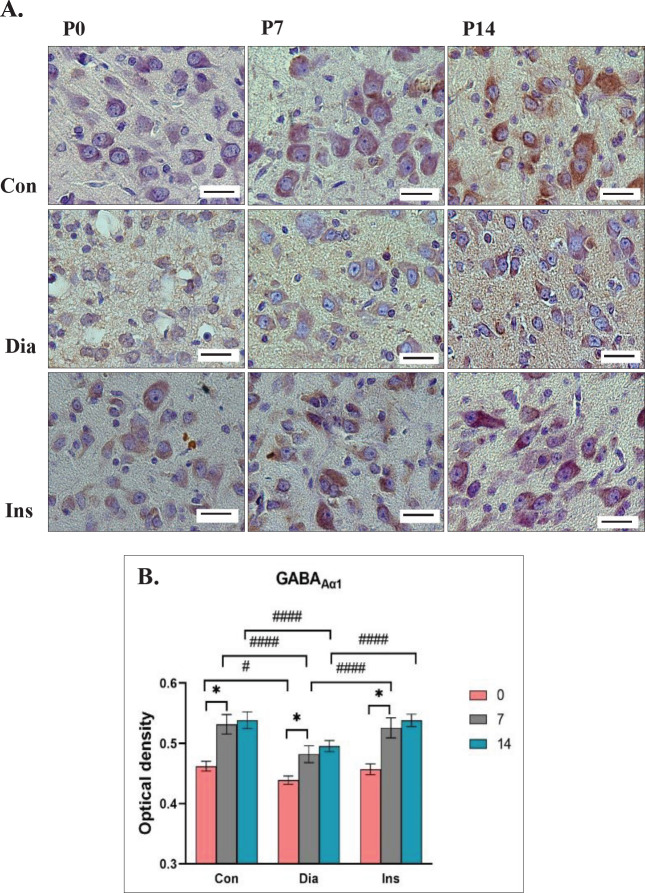
Immunoreactivity of the GABAAα1 in the Lateral Geniculate Body (LGB) which has been displayed by the brown color is different on different days at P0, P7, and P14 in different groups (Con: neonates of control rats, Dia: neonates of diabetic rats, Ins: neonates of diabetic rats treated with insulin)

The main effect on the expression of GABA _Aα1_ in treatment groups showed a significant difference [F (2, 36)=46.89, *P*<0.0001]. In the Dia group, the OD of the GABA_Aα1_ expression was remarkably decreased at P0, P7, and P14 compared with the Con group (*P*<0.05 for P0 and *P*<0.0001 for P7 and P14). The expression of GABA_Aα1_ increased in LGB of rat neonates in the Ins group compared with the Dia group at P7 and P14 (each, *P*<0.0001). A remarkable difference was not detected in the expression of GABA_Aα1_ between rat neonates of the Ins and Con groups at P0, P7, and P14. In all three groups, the OD of GABA_Aα1_ receptor expression in the LGB significantly increased at P7 compared with P0 (*P*<0.0001). The main effect was significantly different in postnatal days [F (2, 36)=152.5, *P*<0.0001]. The highest OD was for the Con group at P14 (0.4723±0.005306), and the lowest OD was for the Dia group at P0 (0.4426±0.005288). A significant difference was not seen in the main interaction effect of treatment groups× postnatal days [F (4, 36)=2.372, *P*=0.0705] (Figure 3).

***Expression of mGluR2 in the LGB***

**Figure 4:**
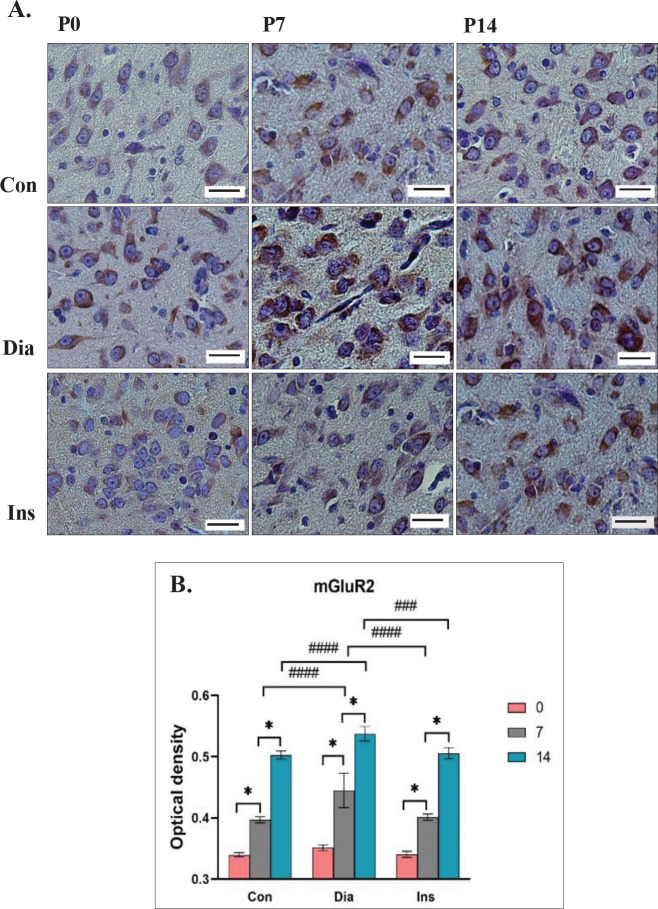
Immunoreactivity of the mGluR2 in the Lateral Geniculate Body (LGB) which has been displayed by the brown color is different on different days at P0, P7, and P14 in different groups (Con: neonates of control rats, Dia: neonates of diabetic rats, Ins: neonates of diabetic rats treated with insulin)

The results of the present study demonstrated that the main effect on the expression of mGluR2 in treatment groups was significantly different [F (2, 36)=35.17, *P*<0.0001]. The expression of mGluR2 in the Dia group remarkably increased compared with the Con group at P7 and P14 (each, *P*<0.0001). In the Ins group, expression of mGluR2 significantly decreased compared with the Dia group at P7 and P14 (*P*<0.0001 and *P*<0.001, respectively). No remarkable difference was detected in the expression of this receptor at P0, P7, and P14 between rat neonates of the Ins and the Con groups. The main effect was significantly different on postnatal days [F (2, 36)=862.3, *P*<0.0001]. In all three groups, the OD of mGluR2 receptor expression in the LGB significantly increased from P0 to P7 and from P7 to P14 (each, *P*<0.0001). No significant difference was found in the main interaction effect of treatment groups× postnatal days [F (4, 36)=4.018, *P*=0.0085]. The highest OD expression was for the Dia group at P14 (0.4445±0.05368) and the lowest OD expression was for the Con group at P0 (0.4131±0.04770) (Figure 4).

AP: Anterior pretectal nucleus, LP: Lateral posterior nucleus of the thalamus, VPM: Ventral posteromedial nucleus of the thalamus, VG: Ventral geniculate nucleus, SubG: sub Geniculate nucleus, CP: Cerebral peduncle, DG: Dentate gyrus, CA: Curnu ammon

The expression of GABAAα1 was significantly decreased at P0, P7, and P14 in the Dia group compared to the Con group. No remarkable difference was perceived in the expression of this receptor in the LGB between rat neonates of the Ins and the Con groups at P0, P7, and P14. Immunoreaction visualization with DAB, counterstaining with Harris hematoxylin; scale bars=100 μm (A). Immunohistochemically staining intensity of GABAAα1 was expressed as Optical Density. Values represent the mean±SD. The level of significance between groups showed by # (# and #### mean *P<*0.05 and *P<*0.0001, respectively). The level of significance between postnatal days showed by * (* means *P<*0.0001) (B)

The expression of mGluR2 was significantly increased at P7 and P14 in the Dia group compared to the Con group. No remarkable difference was perceived in the expression of this receptor in the LGB between rat neonates of the Ins and the Con groups at P0, P7, and P14. Immunoreaction visualization with DAB, counterstaining with Harris hematoxylin; scale bars=100 μm (A). Immunohistochemically staining intensity of mGluR2 was expressed as Optical Density. Values represent the mean±SD. The level of significance between groups showed by # (### and #### mean *P<*0.001 and *P<*0.0001, respectively). The level of significance between postnatal days showed by * (* means *P<*0.0001) (B)

## Discussion

Diabetes in pregnancy has detrimental consequences on the development of the CNS in children (1). Visual impairment and vision loss are among the consequences of diabetes in individuals (30). Diabetes usually leads to neural impairment and also leads to changes in the expression of neurotransmitters (13), including GABA and glutamate content (31), which have an essential role in visual functions in LGB. In the visual system, GABA-containing inhibitory interneurons in the LGN innervate thalamocortical relay neurons by axodendritic and dendrodendritic synapses, also mGluR-mediated actions influence thalamic relay neurons (32). In diabetes, changes in GABA content and glutamate may be related to visual dysfunctions (31). Since no research has been found on the alteration of GABAergic and glutamatergic receptors expression in the visual system of neonates of diabetic mothers, in this study we examined GABA_Aα1,_ GABA_B1,_ and mGluR2 in the LGB of neonates born to diabetic mothers.

***Effects of maternal diabetes on the expression of GABA receptors in the LGB of neonates***

The development of the central nervous system depends on a number of crucial functions performed by GABAergic signaling. There is evidence to suggest that the variety of GABAergic signaling expands during postnatal development to provide the variety and accuracy of inhibitory processes needed for the growing demands of the maturing brain (33). The lateral geniculate nucleus is responsible for transmitting visual information from the retina to the visual cortex. Several modulatory inputs, including GABAergic inputs, affect how this information is processed. Local interneurons within the LGB and extrathalamic projections from the thalamic reticular nucleus are both sources of GABAergic modulation. Through the activation of GABA_A_ and GABA_B_ receptors, these inputs can modify the characteristics of the receptive field and regulate visual attention. GABA receptor heterogeneity’s significance for brain function is still not fully understood (25). In a study on patients with schizophrenia, Jong H. Yoon et al. found that schizophrenia is accompanied by impaired visual inhibition. They discovered lower GABA concentrations in schizophrenia patients’ visual cortex, demonstrating both a deficit in GABA concentrations and a correlation between GABA levels and a behavioral measure of visual inhibition (34). Also, it is possible that in our study, GABA reduction in the LGB following diabetes also reduces visual-related symptoms. Since no studies similar to our work were found, a relationship between diabetes and the expression of GABA receptors in brain regions including the hippocampus has been demonstrated. A study (2019) reported that diabetes reduced GABA receptors expression in the hippocampus of rats. GABA receptor treatment improves the effects of blood glucose on weight gain, blood glucose, and the structure of neurons in the hippocampus (35). Biju *et al*. (1998) also reported that in the brain regions of adult rats inhibitory neurotransmitters (GABA) decreased (36). Also, the results of the present study showed a decrease in the expression of GABA_Aα1 _and GABA_B1_ in the LGB of neonates of diabetic rats compared with the control group. Numerous studies have shown maternal diabetes may lead to CNS development disorders, which may cause changes in many developmental proceedings such as neurogenesis, migration, differentiation, and cell survival, suggesting that maternal diabetes results in neuropathology through numerous mechanisms (37). Diabetes also declines the neuroprotective function of GABAergic neurons in the cerebral cortex, which causes an increase in neuronal damage in the cerebral cortex during hyperglycemia (13). Diabetes decreases antioxidant levels and concurrently rises the production of free radicals (38). Free radicals such as reactive oxygen species (ROS) can affect ionic homeostasis and neurotransmission. Neurotransmission of GABA is sensitive to ROS (39). The effects of ROS on neurotransmission can happen through numerous mechanisms (40). For example, ROS may interact with neurotransmitter receptors and ion transport channels, causing alterations in receptor activity and ionic homeostasis. The sensitivity of inhibitory neurotransmission, including GABA_A_, to ROS, is important because changes in GABA neurotransmission can alter the amount of neuronal excitability and it can be one of the reasons for the reduction of GABA following diabetes (38).

GABAergic synaptogenesis is seen in numerous brain areas, and these neurotransmitters have a trophic role through CNS development. In addition, GABA_A_ receptors may provide the stimulus required for growth and differentiation in the early stages of development. GABA exerts its trophic effect during development through depolarizing action (41). In the present study, the reduction of GABA receptors in the LGB of offspring born to diabetic mothers may lead to a reduction of the trophic role of GABA. On the other hand, insulin therapy modulated these changes in the GABA receptor expression in newborns by lowering blood glucose in diabetic mothers. 

***Effects of maternal diabetes on the expression of mGluR2 receptors in the LGB of neonates***

Metabotropic glutamate presynaptic receptors act as autoreceptors throughout the CNS to inhibit glutamate release and reduce glutamate transfer (42). Glutamate has a dual effect on cell survival in the CNS. Low concentrations of glutamate have trophic effects and enhance neuronal survival and synapse formation, while high concentrations are neurotoxic. The neurotoxicity of glutamate is related to calcium signaling (43). Almost every glutamate receptor subtype is involved in excitotoxic cell death. Different receptor subtypes regulate different signal transduction mechanisms that lead to apoptosis (43). Activation of mGluRs changes geniculate relay cell activity by the depolarization of these cells seen during *in vitro* investigations. Such membrane depolarization controls the activation of a voltage-dependent Ca^2+^ channel. It appears the effect of mGluRs on relay cells in the lateral geniculate nucleus is activated only by cortical inputs. (44). Gestational diabetes mellitus (GDM) stimulates microglial activation and chronic inflammatory responses in the brain of the offspring (45). On the other hand, by stimulating microglial activation, mGluR2 expression increased (46). This is probably one of the reasons for the increase in mGluR2 in neonates born to diabetic mothers. An association between diabetes and mGlu receptor expression has been observed in some brain regions, including the hippocampus. Liu, Zh *et al*. also stated that following the induction of diabetes in the hippocampus, the glutamate content increased, and also the mGluR2/3 subtype receptors are overstimulated. Stimulation of mGluR2/3 suppresses the downstream extracellular signal-regulated kinase (ERK) signaling pathway. ERK modulates downstream pathways that regulate apoptosis. As a result, stimulation of mGluR2-3 decreases ERK expression following diabetes, resulting in the inhibition of apoptotic regulators, and subsequently inducing apoptosis and nerve damage (18). Increased glutamate content is reported to cause neuronal degeneration (47). A study (2004) reported that glutamate and LCCG-I, a specific agonist of the mGluR2 group, reduced the viability of anterior pituitary cells and caused apoptosis in anterior pituitary cells by activating mGluR2 (43). mGluRs can rise cytosolic Ca^2+^ stores in neurons. The mGluR-mediated increase in cytosolic Ca^2+^ concentrations can activate calcium-sensitive K^+^ channels and calcium-dependent non-selective cation channels (48). Ca^2+^ is the major second messenger that supports transmitting depolarization status and synaptic activity to a neuron’s biochemical machine. Ca^2+^ regulation plays a vital role in neurons with extensive and complex signaling pathways (49). In neurons, an increase in local and intracellular Ca^2+^ can play an etiological role in neuronal death (50). Also, the present study showed an increase in the expression of mGluR2 in the LGB of neonates of diabetic mothers compared with the control group. This increase in mGluR2 expression may cause an increase in intracellular calcium concentration and neuronal death which can lead to visual symptoms in the future. In the present study, the expression of mGluR2 was decreased in the LGB of rat neonates born to diabetic mothers treated with insulin due to the lowering of blood glucose levels.

## Conclusion

According to the results of the present study, it can be concluded that diabetes reduces the expression of GABA_Aα1_ and GABA_B1_ receptors but increases the expression of mGluR2 in LGB of male neonates born to diabetic mothers. Due to the important role of these receptors in the visual pathway, changes in the expression of these receptors may cause adverse effects on the visual pathway of neonates of diabetic mothers. Insulin therapy in diabetic mothers can modulate the adverse effects of maternal diabetes in neonates.

## Authors’ Contributions

H H and S F designed this study. N A, F T, and J B have made substantial contributions to data acquisition and/or analysis and interpretation of data. H H, N A, G S, H B helped draft the manuscript or revise it critically for important intellectual content.

## Conflicts of Interest

No potential conflicts of interest were disclosed. 

## References

[1] Lotfi N, Hami J, Hosseini M, Haghir D, Haghir H (2016). Diabetes during pregnancy enhanced neuronal death in the hippocampus of rat offspring. Int J Dev Neurosci.

[2] Ma WX, Tang J, Lei ZW, Li CY, Zhao LQ, Lin C (2020). Potential biochemical mechanisms of brain injury in diabetes mellitus. Aging Dis.

[3] Fleming MD, Benca RM, Behan M (2006). Retinal projections to the subcortical visual system in congenic albino and pigmented rats. Neuroscience.

[4] LeVere T (1978). The primary visual system of the rat: A primer of its anatomy. Physiologic Psychol.

[5] Jonak K, Krukow P, Jonak KE, Radzikowska E, Baj J, Niedziałek A (2020). Decreased volume of lateral and medial geniculate nuclei in patients with LHON disease-7 tesla MRI study. J Clin Med.

[6] Kvale I, Fosse V, Fonnum F (1983). Development of neurotransmitter parameters in lateral geniculate body, superior colliculus and visual cortex of the albino rat. Dev Brain Res.

[7] d’Almeida OC, Violante IR, Quendera B, Moreno C, Gomes L, Castelo-Branco M (2020). The neurometabolic profiles of GABA and Glutamate as revealed by proton magnetic resonance spectroscopy in type 1 and type 2 diabetes. PloS One.

[8] Chebib M, Johnston GA (1999). The ‘ABC’ of GABA receptors: A brief review. Clin Exp Pharmacol Physiol.

[9] Terunuma M (2018). Diversity of structure and function of GABAB receptors: A complexity of GABAB-mediated signaling. Proc Jpn Acad Ser B Phys Biol Sci.

[10] Binns K, Salt T (1997). Different roles for GABAA and GABAB receptors in visual processing in the rat superior colliculus. J Physiol.

[11] Nutt D (2006). GABA A receptors: Subtypes, regional distribution, and function. J Clin Sleep Med.

[12] Papasergi-Scott MM, Robertson MJ, Seven AB, Panova O, Mathiesen JM, Skiniotis G (2020). Structures of metabotropic GABA B receptor. Nature.

[13] Antony S, Peeyush Kumar T, Kuruvilla KP, George N, Paulose C (2010). Decreased GABA receptor binding in the cerebral cortex of insulin induced hypoglycemic and streptozotocin induced diabetic rats. Neurochem Res.

[14] Russell JW, Anjaneyulu M, Berent-Spillson A (2008). Metabotropic glutamate receptors (mGluRs) and diabetic neuropathy. Curr Drug Targets.

[15] Kim J-H, Marton J, Ametamey SM, Cumming P (2020). A review of molecular imaging of glutamate receptors. Molecules.

[16] Mazzitelli M, Palazzo E, Maione S, Neugebauer V (2018). Group II metabotropic glutamate receptors: Role in pain mechanisms and pain modulation. Front Mol Neurosci.

[17] Govindaiah G, Venkitaramani DV, Chaki S, Cox CL (2012). Spatially distinct actions of metabotropic glutamate receptor activation in dorsal lateral geniculate nucleus. J Neurophysiol.

[18] Liu Z, Han Y, Zhao H, Luo W, Jia L, Wang Y (2019). Glu-mGluR2/3-ERK signaling regulates apoptosis of hippocampal neurons in diabetic-depression model rats. Evid Based Complement Alternat Med.

[19] Chen H, Wang M, Xia L, Dong J, Xu G, Wang Z (2022). New evidence of central nervous system damage in diabetes mellitus: Impairment of fine visual discrimination. Diabetes.

[20] Lau JC, Kroes RA, Moskal JR, Linsenmeier RA (2013). Diabetes changes expression of genes related to glutamate neurotransmission and transport in the Long-Evans rat retina. Mol Vis.

[21] Furman BL (2021). Streptozotocin-induced diabetic models in mice and rats. Curr Protoc Pharmacol.

[22] Rezazadeh H, Sharifi MR, Sharifi M, Soltani N (2021). Gamma-aminobutyric acid attenuates insulin resistance in type 2 diabetic patients and reduces the risk of insulin resistance in their offspring. Biomed Pharmacother.

[23] Abbasi F, Baradaran R, Khoshdel-Sarkarizi H, Kargozar S, Hami J, Mohammadipour A (2020). Distribution pattern of nicotinic acetylcholine receptors in developing cerebellum of rat neonates born of diabetic mothers. J Chem Neuroanat.

[24] Haghir H, Rezaee AAR, Sankian M, Kheradmand H, Hami J (2013). The effects of induced type-I diabetes on developmental regulation of insulin & insulin like growth factor-1 (IGF-1) receptors in the cerebellum of rat neonates. Metab Brain Dis.

[25] Ye Z, Yu X, Houston CM, Aboukhalil Z, Franks NP, Wisden W (2017). Fast and slow inhibition in the visual thalamus is influenced by allocating GABAa receptors with different γ subunits. Front Cell Neurosci.

[26] Vahidinia Z, Alipour N, Atlasi MA, Naderian H, Beyer C, Azami Tameh A (2017). Gonadal steroids block the calpain-1-dependent intrinsic pathway of apoptosis in an experimental rat stroke model. Neurol Res.

[27] Paxinos G, Watson C The Rat Brain in Stereotaxic Coordinates 2005; San Diego.

[28] Ramachandra R, Subramanian T (2016). Atlas of the neonatal rat brain.

[29] Carpi-Santos R, Maggesissi R, von Seehausen M, Calaza K (2017). Retinal exposure to high glucose condition modifies the GABAergic system: Regulation by nitric oxide. Exp Eye Res.

[30] Santiago AR, Gaspar JM, Baptista FI, Cristóvão AJ, Santos PF, Kamphuis W (2009). Diabetes changes the levels of ionotropic glutamate receptors in the rat retina. Mol Vis.

[31] Honda M, Inoue M, Okada Y, Yamamoto M (1998). Alteration of the GABAergic neuronal system of the retina and superior colliculus in streptozotocin-induced diabetic rat. Kobe J Med Sci.

[32] Govindaiah G, Cox CL (2006). Metabotropic glutamate receptors differentially regulate GABAergic inhibition in thalamus. J Neurosci.

[33] Perreault MC, Qin Y, Heggelund P, Zhu JJ (2003). Postnatal development of GABAergic signalling in the rat lateral geniculate nucleus: presynaptic dendritic mechanisms. J Physiol.

[34] Yoon JH, Maddock RJ, Rokem A, Silver MA, Minzenberg MJ, Ragland JD (2010). GABA concentration is reduced in visual cortex in schizophrenia and correlates with orientation-specific surround suppression. J Neurosci.

[35] Tu LL, Sun Q, Wei LL, Shi J, Li JP (2019). Upregulation of GABA receptor promotes longterm potentiation and depotentiation in the hippocampal CA1 region of mice with type 2 diabetes mellitus. Exp Ther Med.

[36] Biju M, Paulose C (1998). Brain glutamate dehydrogenase changes in streptozotocin diabetic rats as a function of age. Biochem Mol Biol Int.

[37] Sadeghi A, Esfandiary E, Hami J, Khanahmad H, Hejazi Z, Razavi S (2016). Effect of maternal diabetes on gliogensis in neonatal rat hippocampus. Adv Biomed Res.

[38] Sah R, Galeffi F, Ahrens R, Jordan G, Schwartz-Bloom RD (2002). Modulation of the GABAA-gated chloride channel by reactive oxygen species. J neurochem.

[39] Muriach M, Flores-Bellver M, Romero FJ, Barcia JM (2014). Diabetes and the brain: Oxidative stress, inflammation, and autophagy. Oxid Med Cell Longev.

[40] Van der Vliet A, Bast A (1992). Effect of oxidative stress on receptors and signal transmission. Chem Biol Interact.

[41] Ikonomovic S, Kharlamov E, Manev H, Ikonomovic MD, Grayson DR (1997). GABA and NMDA in the prevention of apoptotic-like cell death in vitro. Neurochem Int.

[42] Chen CY, Ling Eh, Horowitz JM, Bonham AC (2002). Synaptic transmission in nucleus tractus solitarius is depressed by Group II and III but not Group I presynaptic metabotropic glutamate receptors in rats. J Physiol.

[43] Caruso C, Bottino M, Pampillo M, Pisera D, Jaita G, Duvilanski B (2004). Glutamate induces apoptosis in anterior pituitary cells through group II metabotropic glutamate receptor activation. Endocrinology.

[44] Godwin DW, Vaughan JW, Sherman SM (1996). Metabotropic glutamate receptors switch visual response mode of lateral geniculate nucleus cells from burst to tonic. J Neurophysiol.

[45] Vuong B, Odero G, Rozbacher S, Stevenson M, Kereliuk SM, Pereira TJ (2017). Exposure to gestational diabetes mellitus induces neuroinflammation, derangement of hippocampal neurons, and cognitive changes in rat offspring. J Neuroinflam.

[46] Taylor DL, Jones F, Kubota ESCS, Pocock JM (2005). Stimulation of microglial metabotropic glutamate receptor mGlu2 triggers tumor necrosis factor α-induced neurotoxicity in concert with microglial-derived Fas ligand. J Neurosci.

[47] Balakrishnan S, Kumar P, Paulose C (2009). Glutamate (mGluR-5) gene expression in brain regions of streptozotocin induced diabetic rats as a function of age: role in regulation of calcium release from the pancreatic islets in vitro. J Biomed Sci.

[48] Fagni L, Chavis P, Ango F, Bockaert J (2000). Complex interactions between mGluRs, intracellular Ca2+ stores and ion channels in neurons. Trends Neurosci.

[49] Gleichmann M, Mattson MP (2011). Neuronal calcium homeostasis and dysregulation. Antioxid Redox Signal.

[50] Johnson ME, Gores GJ, Uhl CB, Sill JC (1994). Cytosolic free calcium and cell death during metabolic inhibition in a neuronal cell line. J Neurosci.

